# Interleukin-7 Contributes to the Invasiveness of Prostate Cancer Cells by Promoting Epithelial–Mesenchymal Transition

**DOI:** 10.1038/s41598-019-43294-4

**Published:** 2019-05-06

**Authors:** Min A. Seol, Jin-Hee Kim, Keunhee Oh, Gwanghun Kim, Myung Won Seo, Young-Kyoung Shin, Ji Hyun Sim, Hyun Mu Shin, Bo Yeon Seo, Dong-Sup Lee, Ja-Lok Ku, Ilkyu Han, Insoo Kang, Serk In Park, Hang-Rae Kim

**Affiliations:** 1 Department of Anatomy and Cell Biology, Seoul National University College of Medicine, Seoul, 03080 Republic of Korea; 2 Department of Biomedical Sciences, Seoul National University College of Medicine, Seoul, 03080 Republic of Korea; 3 BK21Plus Biomedical Science Project, Seoul National University College of Medicine, Seoul, 03080 Republic of Korea; 4 Cancer Research Institute, Seoul National University College of Medicine, Seoul, 03080 Republic of Korea; 5 Department of Orthopedic Surgery, Seoul National University College of Medicine, Seoul, 03080 Republic of Korea; 6 Medical Research Institute, Seoul National University College of Medicine, Seoul, 03080 Republic of Korea; 7 Department of Biomedical Laboratory Science, College of Health Science, Cheongju University, Cheongju, Chungbuk 28150 Republic of Korea; 8 Department of Internal Medicine, Section of Rheumatology, Yale University School of Medicine, New Haven, CT 06520 USA; 9 Department of Biochemistry and Molecular Biology, Korea University College of Medicine, Seoul, 02841 Republic of Korea; 10 BK21 Plus Program, Korea University College of Medicine, Seoul, 02841 Republic of Korea; 11 Department of Medicine, Vanderbilt University School of Medicine, Nashville, TN 37232 USA

**Keywords:** Prostate cancer, Bone metastases, Prostate cancer

## Abstract

Precise mechanisms underlying interleukin-7 (IL-7)-mediated tumor invasion remain unclear. Thus, we investigated the role of IL-7 in tumor invasiveness using metastatic prostate cancer PC-3 cell line derivatives, and assessed the potential of IL-7 as a clinical target using a Janus kinase (JAK) inhibitor and an IL-7-blocking antibody. We found that IL-7 stimulated wound-healing migration and invasion of PC-3 cells, increased phosphorylation of signal transducer and activator of transcription 5, Akt, and extracellular signal-regulated kinase. On the other hand, a JAK inhibitor and an IL-7-blocking antibody decreased the invasiveness of PC-3 cells. IL-7 increased tumor sphere formation and expression of epithelial–mesenchymal transition (EMT) markers. Importantly, lentiviral delivery of IL-7Rα to PC-3 cells significantly increased bone metastasis in an experimental murine metastasis model compared to controls. The gene expression profile of human prostate cancer cells from The Cancer Genome Atlas revealed that EMT pathways are strongly associated with prostate cancers that highly express both IL-7 and IL-7Rα. Collectively, these data suggest that IL-7 and/or IL-7Rα are promising targets of inhibiting tumor metastasis.

## Introduction

Prostate cancer is a significant problem in health of men. In the United States, approximately 8% of cancer-related deaths in men are caused by prostate cancer^1^. In particular, bone metastases are very common in the patients with prostate, breast and lung cancers^2^. These patients have a very poor quality of life due to multiple problems, such as pathologic fracture, compression of the spinal cord, hypercalcemia, and extreme pain in the bone, together with a poor prognosis and very low survival rate^3,4^.

Recent studies have shown that interleukin-7 (IL-7) affects tumor cell invasion and growth, whereas IL-7 is classically shown to play critical roles in the production and differentiation of lymphocytes in the thymus and bone marrow and in the survival of naïve and memory T cells in the periphery^5^. For example, IL-7 is associated with the invasiveness of DU-145 prostate cancer cells and 5637 bladder cancer cells, possibly via the nuclear factor-κB (NF-κB) signaling pathway and upregulation of metalloproteinases (MMPs)^6,7^. By inducing cyclin D1 upregulation via the c-Fos/c-Jun pathway, IL-7 promotes the proliferation of lung cancer cells^8^.

Although IL-7 is secreted mainly by stromal cells in the thymus and bone marrow^9^, IL-7 is also dysregulated at the transcriptional level in renal and colorectal cancer cells and is concentrated in the cancer cells, plasma, and tissues of ovarian cancer patients^10–13^. Patients with prostate cancer are more likely to express IL-7 than are those with benign prostatic hyperplasia^14^. The higher the Gleason grade in prostate cancer patients, the higher the activity of signal transducer and activator of transcription 5 (STAT5), a downstream target of IL-7 receptor (IL-7R) signaling^15^. On the other hand, STAT5 is activated in 61% of distant metastases of clinical prostate cancer, and constitutive activation of STAT5 in PC-3 and DU-145 prostate cancer cells increases cell migration and invasion^16^. IL-7, behaving in an autocrine or paracrine manner, plays a critical role in the bone metastasis of solid tumors, such as lung and breast cancer^17–19^.

However, the mechanism of cancer metastasis or growth induced by IL-7 remains controversial, and its role in human clinical conditions is not yet understood. Thus, we explored the mechanism through which IL-7 influences the invasiveness of prostate cancer, and whether the invasiveness induced by IL-7 can be controlled by inhibitors of this mechanism.

In this study, we analyzed PC-3 cells, an androgen-independent osteolytic line derived from bone metastasis of adenocarcinoma^20^. We determined whether tumor cell migration and invasion are specific for IL-7 and analyzed IL-7R signaling and epithelial–mesenchymal transition (EMT)-related molecules. We evaluated the potential of using an IL-7 blocking antibody (Ab) and a Janus kinase (JAK) inhibitor to control IL-7-induced tumor cell migration and invasion. We then validated the mechanism of IL-7-induced tumor cell migration and invasion in prostate cancer using The Cancer Genome Atlas (TCGA) database. The findings of this study provide a basis for the development of effective therapeutic agents for the metastatic prostate cancer treatment.

## Results

### IL-7 enhances the migration and invasion of prostate cancer cells

**Figure 1:**
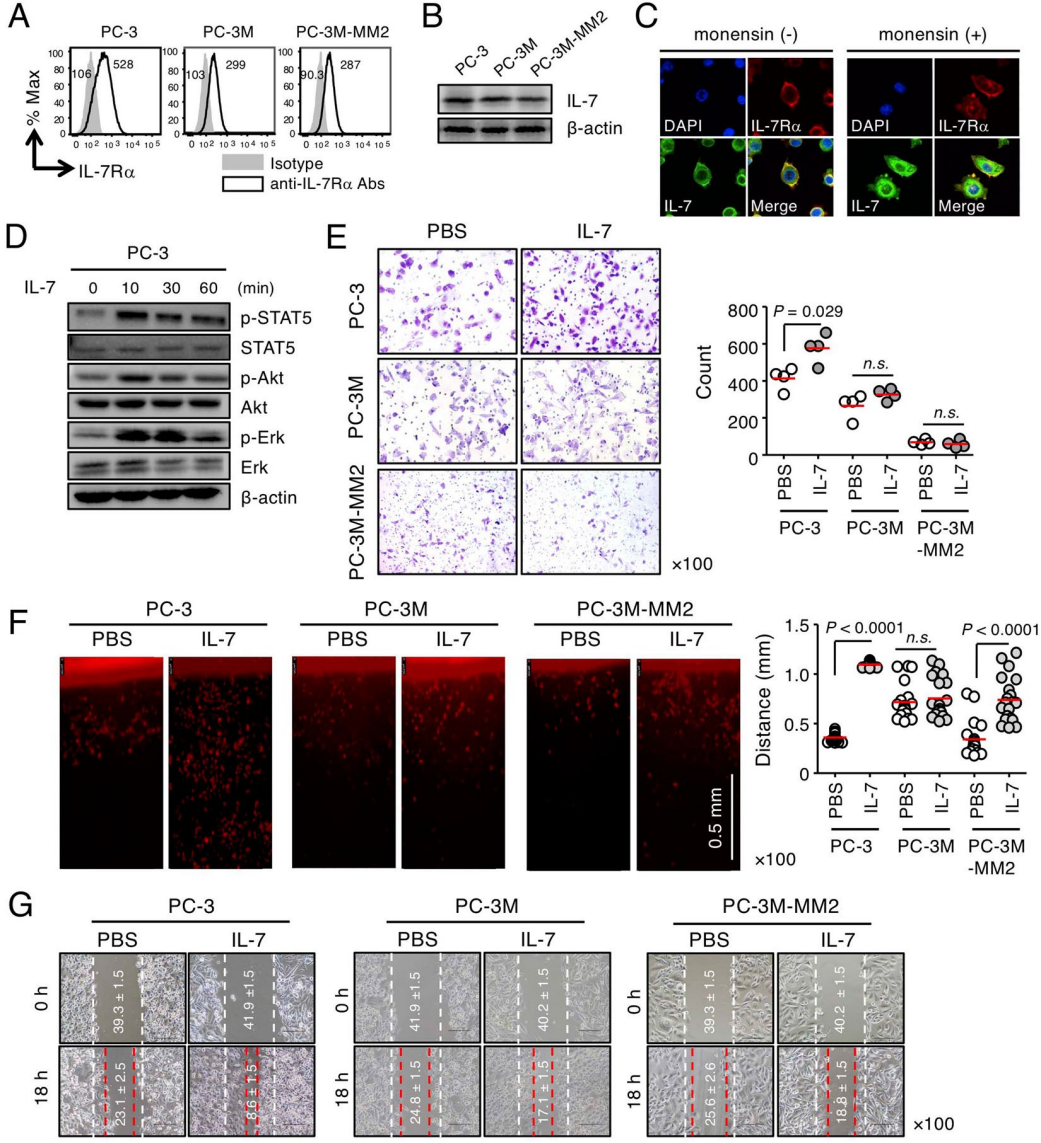
IL-7 responsiveness to PC-3 prostate cancer cells. (**A**) PC-3, PC-3M, and PC-3M-MM2 cells were stained with anti-IL-7Rα Abs and analyzed by flow cytometry. The numbers in the histogram indicate the mean fluorescence intensity. (**B**) Cells were subjected to immunoblot analysis of IL-7 expression. (**C**) Surface IL-7Rα and intracellular IL-7 expression levels in PC-3 cells in the presence or absence of monensin were measured by immunofluorescence. (**D**) PC-3 cells were stimulated with IL-7 (100 ng/mL) for the appropriate times and then subjected to immunoblot analysis of STAT5, Akt, and Erk phosphorylation. (**E**) PC-3, PC-3M, and PC-3M-MM2 cells were left to invade through matrigel (250 μg/mL) for 24 h with or without IL-7 (10 ng/mL). Invading PC-3 cells were stained with crystal violet (left panel), counted using ImageJ software, and plotted as a graph (right panel). (**F**) Dil-labeled PC-3, PC-3M, and PC-3M-MM2 cells were allowed to invade through vertical collagen gel (2 mg/mL) for 18 h with or without of IL-7 (10 ng/mL). Representative images of cells invading vertical collagen gel (left panel) and dot graphs showing the maximum distances of cell invasion (right panel). Scale bar = 0.5 mm. (**G**) Wound-healing migration of PC-3, PC-3M, and PC-3M-MM2 cells was performed after IL-7 (10 ng/mL) treatment for 6 h. Dashed lines indicate the initial boundaries of the scratches (white dotted lines, 0 h) and the cell leading edges at a subsequent time point (red dotted lines, 6 h). The numbers in the picture indicate the average distances of the wound width (mean ± SEM, μm). The bars indicate means. *P*-values were obtained using the Mann–Whitney *U* test (**D–F**). *n*.*s*., not significant. Results represent two or three independent experiments.

We first explored whether prostate cancer cell line, PC-3, and its derivatives express IL-7 and IL-7Rα, and whether IL-7Rα expressed by these cells responds to IL-7. We found that both IL-7 and IL-7Rα were expressed in metastatic androgen-independent PC-3 cells and PC-3-derived cells, PC-3M and PC-3M-MM2 (Fig. 1A,B). PC-3M^21^ and PC-3M-MM2^22^ were established from cells that metastasized in the liver and bone, respectively, by transplanting PC-3 cells into nude mice. In particular, when PC-3 cells were treated with monensin (a protein transport inhibitor), intracellular IL-7 expression increased (Fig. 1C), indicating that PC-3 cells constitutively produce IL-7. After IL-7 binds to its receptor, the interaction between JAK1 and JAK3 leads to phosphorylation of STAT5, a key signaling molecule that regulates gene expression^23,24^. In addition, STAT5 induces phosphatidylinositol 3-kinase (PI3K)/Akt and phosphorylation of MAPKs, such as extracellular-signal-regulated kinase (Erk)^9,25–27^. We found that IL-7 induced the phosphorylation of STAT5, Akt, and Erk in PC-3 cells (Fig. 1D). These data indicate that PC-3 prostate cancer cells act as both a source of IL-7 and target cells that respond to its own or exogenous IL-7. This finding suggests that IL-7-dependent tumorigenesis or metastasis of prostate cancer cells, such as PC-3 cells, is possible but not seen in other tumors^28^.

**Figure 2:**
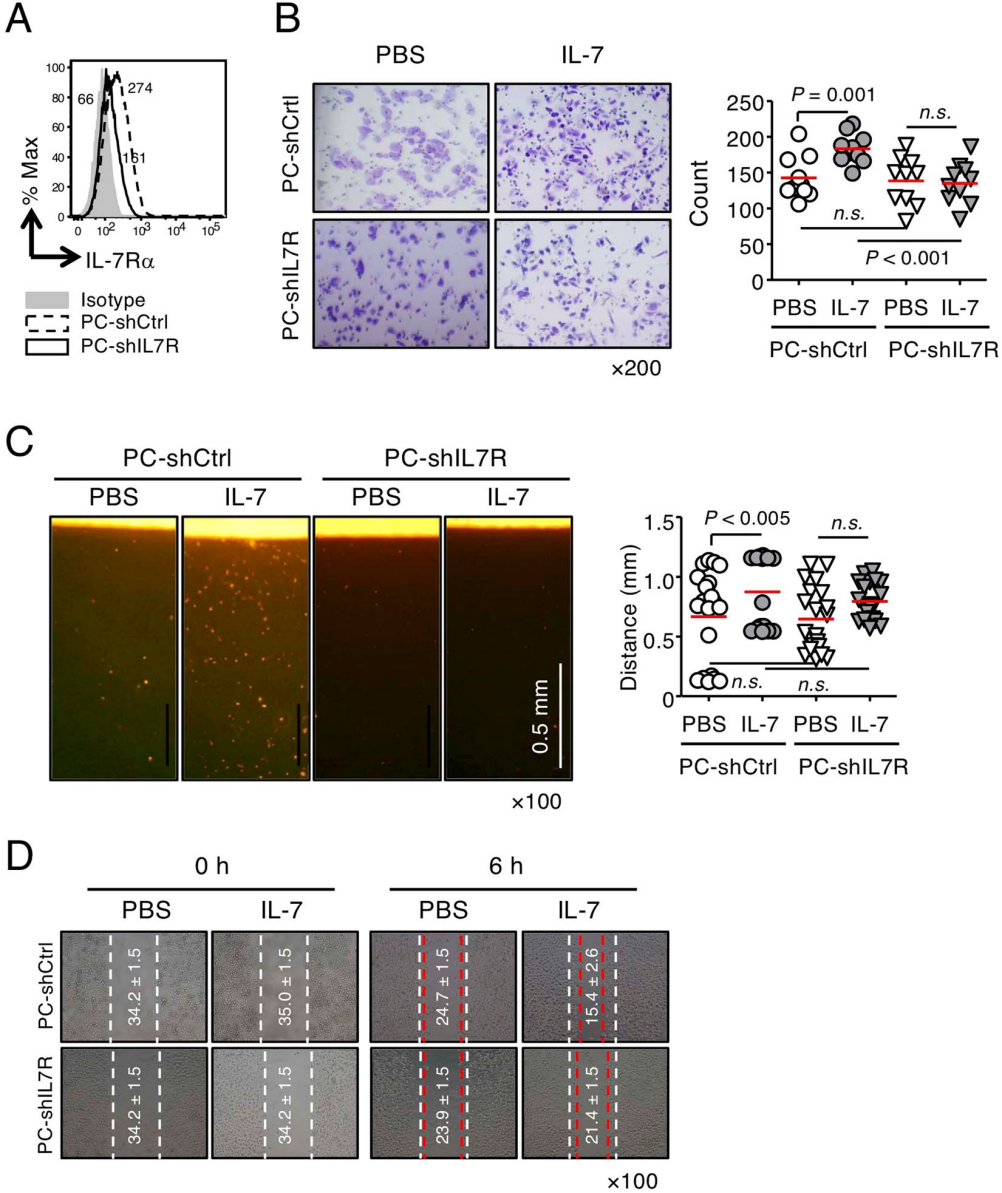
Effects of IL-7 on the migration and invasion of IL-7Rα knockdown PC-3 cells. (**A**) IL-7Rα knockdown PC-3 (PC-shIL7R) and control cells (PC-shCtrl) were established by lentiviral transduction. Cells were stained with anti-IL-7Rα Abs and analyzed by flow cytometry. The numbers in the histogram indicate the mean fluorescence intensity. (**B**) Cells were left to invade through matrigel (250 μg/mL) for 24 h with or without IL-7 (10 ng/mL). Invading cells were stained with crystal violet (left panel), counted using ImageJ software, and plotted as a graph (right panel). (**C**) Dil-labeled cells were allowed to invade vertical collagen gel (2 mg/mL) for 18 h with or without IL-7 (10 ng/mL). Representative image of cells invading vertical collagen gel (left panel) and dot graph showing the maximum distances of cell invasion (right panel). Scale bar = 0.5 mm. (**D**) Wound-healing migration of PC-shIL7R and control cells was performed after IL-7 (10 ng/mL) treatment for 6 h. Dashed lines indicate the initial boundaries of the scratches (white dotted lines, 0 h) and the cell leading edges at a subsequent time point (red dotted lines, 6 h). The numbers in the picture indicate the average distances of the wound width (mean ± SEM, μm). The bars represent means. *P*-values were obtained using the Mann–Whitney *U* test (**B**,**C**). *n*.*s*., not significant. Results represent two or three independent experiments.

Based on these observations, we examined variations in the migration and invasion of PC-3 cells by IL-7 treatment. The Matrigel matrix solution (*i*.*e*., matrigel), which simulates the basement membrane, and collagen^29^, a major component of the extracellular matrix (ECM) that accounts for 90% of bone matrix protein content^30,31^, were used to analyze the invasion of PC-3, PC-3M, PC-3M-MM2 (Fig. 1E,F), PC-shIL7R (knockdown of IL-7Rα, Fig. 2A), and PC-IL7ROE cells (overexpression of IL-7Rα, Supplementary Fig. S1A). Under these artificial ECM conditions, PC-3 cells showed a considerable increase in invasion when stimulated with IL-7 (Fig. 1E,F). Compared with the parent PC-3 cells, PC-3M (Fig. 1E,F) and PC-shIL7R cells were less responsive to IL-7 (Fig. 2B,C); in contrast, PC-IL7ROE cells showed a significant increase in cell invasion regardless of IL-7 treatment (Supplementary Fig. S1B,C). It is possible that the increased invasiveness caused by IL-7Rα overexpression is secondary to the production of IL-7 in an autocrine manner (Fig. 1B,C). However, PC-3M-MM2 cells significantly increased invasion in response to IL-7 only under collagen conditions but not in matrigel (Fig. 1F). In addition, wound healing cell migration was IL-7 dependent (Fig. 1G,D and Supplementary Fig. S1D), similar to the degree of invasiveness observed by cells in matrigel and collagen.

Taken together, these results showed that IL-7 acts on prostate cancer cells expressing IL-7Rα to increase their migration and invasion. In addition, since PC-3 cells can produce IL-7, we believe it is important to control IL-7Rα expression, the interaction between IL-7 and IL-7R, and IL-7Rα signaling to regulate cell invasion.

### Inhibition of IL-7 binding to its receptor or IL-7R downstream signaling significantly decreased the migration and invasion of PC-3 cells

IL-7 directly enhances the migration and invasion of PC-3 cells; thus, we explored the potential of IL-7 as a target for the inhibition of prostate cancer metastasis. First, we used an anti-IL-7 Ab, M25, to inhibit the interaction between IL-7 and IL-7R. M25 markedly inhibited STAT5 phosphorylation in a dose-dependent manner (Supplementary Fig. S2A) and significantly inhibited the invasion of PC-3 cells (Supplementary Fig. S2B,C), indicating suppressed IL-7R signaling. Interestingly, PC-3 cell invasion was repressed by M25 treatment, even in the absence of exogenous IL-7 (Supplementary Fig. S2B,C), indicating that M25 inhibits PC-3 cell basal invasion via endogenous IL-7, as proposed in our previous results (Figs 1 and 2 and Supplementary Fig. S1).

**Figure 3:**
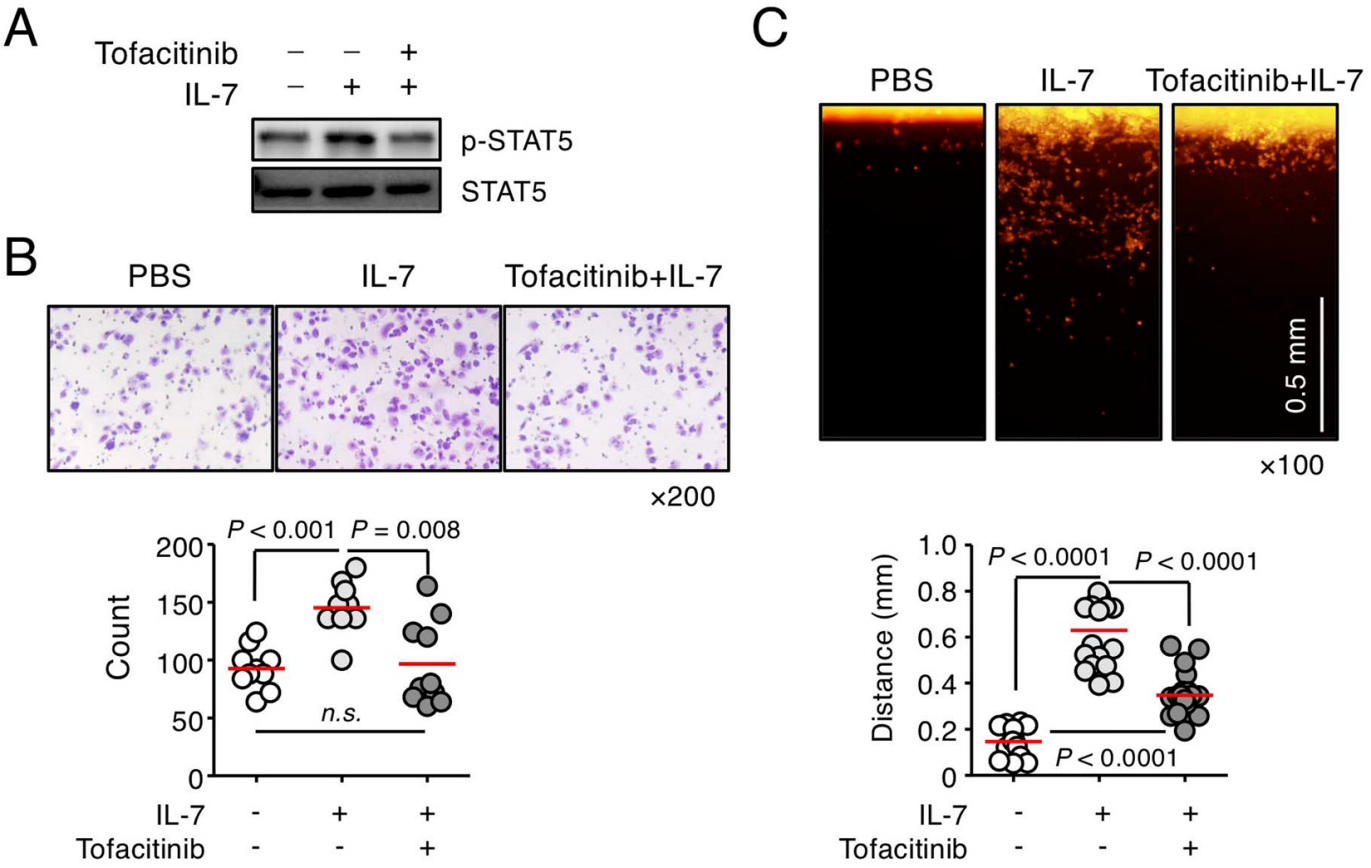
A JAK inhibitor, tofacitinib, suppressed the invasiveness of PC-3 cells induced by IL-7. (**A**) PC-3 cells were treated with a JAK inhibitor, tofacitinib (100 ng/mL), for 1 h, followed by IL-7 (100 ng/mL) treatment for 30 min, and were then subjected to immunoblot analysis of STAT5 phosphorylation. (**B**) PC-3 cells were left to invade through matrigel (250 μg/mL) for 24 h after treatment with IL-7 (10 ng/mL) with or without tofacitinib (100 ng/mL). Invading cells were stained with crystal violet (upper panel), counted using ImageJ software, and plotted as a graph (lower panel). (**C**) Dil-labeled cells were left to invade through vertical collagen gel (2 mg/mL) for 18 h after treatment with IL-7 (10 ng/mL) with or without of tofacitinib (100 ng/mL). Representative image of cells invading vertical collagen gel (upper panel) and dot graph showing the maximum distances of the invading cells (lower panel). Scale bar = 0.5 mm. Bars indicate means. *P*-values were done by the Mann–Whitney *U* test (**B**,**C**). Results represent two or three independent experiments.

Furthermore, to explore the clinical utility of IL-7R signaling, we used tofacitinib, a JAK inhibitor, which interferes with the JAK/STAT signaling pathway, for the treatment of rheumatoid arthritis^32–34^. In response to IL-7, tofacitinib inhibited the phosphorylation of STAT5 in PC-3 cells (Fig. 3A) and strongly inhibited the IL-7-induced invasion of PC-3 cells (Fig. 3B,C). These findings suggest that IL-7 and IL-7R could be target molecules for the treatment of prostate cancer metastasis.

### IL-7 induces epithelial–mesenchymal transition and promotes metastasis, but does not affect tumorigenesis or growth of PC-3 cells

We next examined the mechanism via which IL-7 increases the migration and invasion of PC-3 cells. Prior to addressing this question, we performed an *in vitro* proliferation assay of PC-3 cells, since IL-7 influences the proliferation of lung cancer cells by modulating cyclin D1, a cell-cycle regulator^8^. Unexpectedly, we found no difference in IL-7-induced proliferation between PC-3 cells and PC-IL7ROE cells (Supplementary Fig. S3A). When PC-CtrlOE and PC-IL7ROE cells were subcutaneously injected into mice, both tumor cells began to grow at similar times, with no significant difference in growth rate (Supplementary Fig. S3B). To exclude the effect of the IL-7 source on tumor growth in mice, we examined the effects of IL-7 from mice on human PC-3 cells. Our results show that PC-3 cells respond to IL-7 derived from either mice or humans Supplementary Fig. S4). Taken together, these findings demonstrated that IL-7 does not augment tumorigenesis or tumor growth, despite promoting invasion and migration of PC-3 cells.

Meanwhile, MMPs have a critical effect on the metastatic process of tumor cells because of their ability to hydrolyze proteins^35,36^. For example, the gelatinases MMP2 and MMP9 affect bone matrix turnover and increase bone mineral density in prostate cancer^37,38^. MMP1 and MMP13, which are collagenases, and MMP7, a matrilysin, are highly expressed in metastatic prostate cancer^39^ and increase the activity of osteoclasts^40–42^. Based on these observations, we measured the mRNA levels and enzyme activities of MMPs after treating PC-3 cells with IL-7. We observed no differences in the mRNA expression of MMPs after IL-7 treatment, even in PC-IL7ROE cells (Supplementary Fig. S5A), or in the enzymatic activities of MMP2 and MMP9 based on gelatin zymography (Supplementary Fig. S5B). Thus, the increase in migration and invasion by IL-7 may be promoted by factors other than MMPs.

**Figure 4:**
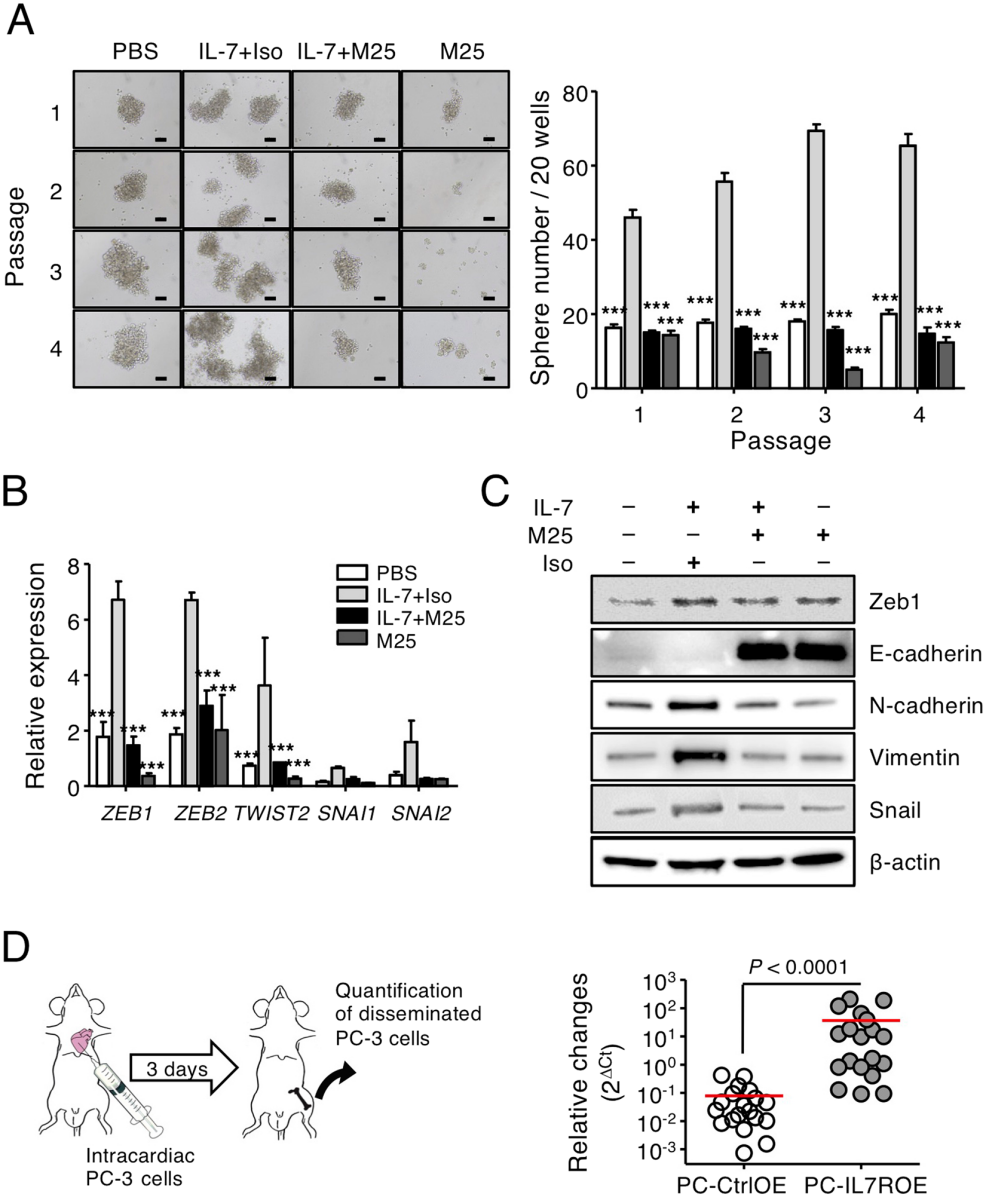
IL-7 induced the epithelial–mesenchymal transition in PC-3 cells and promotes metastasis of PC-3 cells to bone. (**A**) For the sphere formation assay, PC-3 cells were cultured on nonadherent 96-well plates at 2 × 10^2^ /100 μL/well (n = 20) and given an IL-7 (100 ng/mL) treatment in the presence of M25 or isotype control Abs (Iso) (100 μg/mL) for 7 days. Visible sphere counts were done under a microscope (left panel) and plotted as a graph (right panel). Original magnification, ×10. Scale bar = 100 μm. Results are the averages from three independent experiments. (**B**) The transcriptional levels of *ZEB1*, *ZEB2*, *TWIST1*, *SNAI1*, and *SNAI2* in PC-3 cells after IL-7 treatment (10 ng/mL) in the presence of M25 or isotype control Abs (Iso) (100 μg/mL) for 24 h were measured by quantitative RT-PCR. The graph shows the relative gene expression levels normalized to *GAPDH*. Results are representative of four independent experiments. *P*-values were done by one-way ANOVA, followed by Bonferroni *post hoc* tests; ***P < 0.001 (***) *vs*. IL-7-treated PC-3 cells (**A**,**B**). (**C**) The protein levels of Zeb1, E-cadherin, N-cadherin, vimentin, and Snail were measured in PC-3 cells after IL-7 treatment (10 ng/mL) for 72 h. Results are representative of three independent experiments. (**D**) To assess skeletal metastasis, qPCR was used to compare metastasis of PC-CtrlOE and PC-IL7ROE cells (right panel) after intra-cardiac injection of cells (left panel) as described in the Materials and Methods (n = 19 each). The relative changes in metastasis of tumor cells were calculated as 2^−ΔCt^. The bars indicate means. *P*-values were obtained using the Mann–Whitney *U* test.

In this regard, we noticed that EMT, characterized by a progressive loss of epithelial markers^43^, causes proteolysis and increases the motility of tumor cells^44^. In addition, induction of EMT in neoplastic cell populations results in increased cell populations with stem-like properties^45^, while cancer stem cells (CSCs) are strongly associated with the phenotypic characteristics observed during the induction of EMT in cancer cells. Thus, sphere-forming ability was evaluated as an indicator of EMT and CSCs^46,47^. We found that IL-7 treatment significantly increased the sphere formation of PC-3 cells, whereas M25 suppressed this effect, even in the absence of exogenous IL-7 (Fig. 4A). The self-renewal capacity of PC-3 by IL-7 was also maintained even after serial passages (Fig. 4A). Consistent with the findings in the wound-healing cell migration and invasion assays, treating PC-3 cells with IL-7 significantly increased the transcription of EMT-related genes^44,48,49^, such as *ZEB1*, *ZEB2*, and *TWIST1*, but did not significantly increase the transcription of *SNAI1* and *SNAI2* (Fig. 4B). Indeed, *ZEB1* and *ZEB2* mRNA, highlighted on promoting EMT^50–53^, were expressed at >4-fold greater levels in PC-3 cells stimulated with IL-7 stimulation compared to the control (Fig. 4B). The increased transcription of EMT-related genes induced by IL-7 returned to basal levels following M25 treatment (Fig. 4B). During EMT, E-cadherin (a marker of epithelial cells) levels decrease, and N-cadherin, Zeb1 and vimentin (markers of mesenchymal cells) levels increase^48,54^. Although E-cadherin was originally expressed at a low level in PC-3 cells, it was elevated above the basal level after M25 treatment with or without IL-7 (Fig. 4C). We found that that protein expression of N-cadherin, vimentin, Zeb1, and Snail was increased in PC-3 cells by IL-7 stimulation, whereas their expression decreased after M25 treatment (Fig. 4C). The expression of EMT markers was also affected by treatment with M25 alone, presumably because PC-3 cells constitutively produce IL-7 during culture (Fig. 1). Consistent with the tumor cell migration and invasion effects by M25 treatment (Fig. 2B,C), these results suggest that basal expression of EMT-related molecules in PC-3 cells may be caused by self-produced IL-7.

To determine whether IL-7 promotes bone metastasis *in vivo*, we injected PC-3 cells into the systemic circulation in athymic nude mice via intra-cardiac injection^55^ (Fig. 4D). In particular, we were able to evaluated early-onset tumor dissemination in bone using *Alu*-based quantification, as previously described^56,57^. Interestingly, the relative amount of disseminated tumor cells was significantly higher in the PC-IL7ROE than the control of PC-CtrlOE injected mice (Fig. 4D), indicating that PC-IL7ROE promoted the development of experimental bone metastasis. These findings suggest that IL-7 augments the migration and invasion of PC-3 cells via EMT.

### GSEA shows enrichment of EMT-related genes in IL-7Rα^high^IL-7^high^ patients

**Figure 5:**
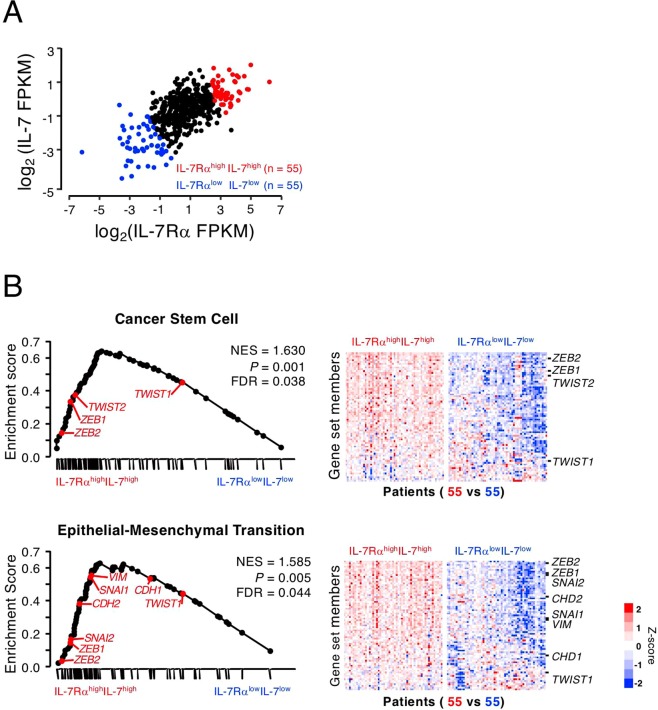
IL-7- and IL-7Rα-expressing prostate cancers showed enrichment of epithelial–mesenchymal transition (EMT) and cancer stem cells gene sets. Public data sets for prostate cancer samples (n = 551) were collected from The Cancer Genome Atlas and subjected to gene set enrichment analysis (GSEA). (**A**) The patient gene expression profiles were categorized after correlating IL-7Rα and IL-7 expression distributions. The top and bottom 10% of the population were defined as the IL-7Rα^high^ IL-7^high^ and IL-7Rα^low^ IL-7^low^ groups (n = 55 each), respectively. (**B**) GSEA plots indicate significant enrichment of cancer stem cells and EMT in the IL-7Rα^high^IL-7^high^ compared with IL-7Rα^low^IL-7^low^ patient groups. Genes were ordered according to their ranked ratios, and GSEA was performed using the GSEA tool at http://www.broad.mit.edu/gsea. The plot (black curve) shows the enrichment score (ES), a running-sum statistic, for ranked genes compared with cancer stem cells (upper panel) and EMT (lower panel) gene set. The normalized enrichment scores (NESs) and nominal *P*-values are indicated. Colors are mRNA expression levels of each gene normalized by the Z score transformation.

Based on the cell line results, we explored whether the expression levels of IL-7 and IL-7Rα are associated with the expression of EMT-related genes in cancer patient samples. We performed GSEA of TCGA data from 551 samples from 499 patients with prostate cancer. Interestingly, there was a statistically significant correlation between IL-7Rα and IL-7 expression in prostate cancer (Fig. 5A, r = 0.63, *P* < 0.001), suggesting that expression of the two molecules occurs simultaneously. We divided the population into IL-7Rα^high^IL-7^high^ and IL-7Rα^low^IL-7^low^ groups according to IL-7 and IL-7Rα expression levels (top 10% and bottom 10%, respectively). The two groups showed significant differences in the expression of stemness-related genes (*e*.*g*., CSC and stem cell signaling), metastasis-related genes (*e*.*g*., EMT and cell motility), and signaling pathway genes (*e*.*g*., *IL6/STAT3*, *TNF*, *NFKB*, *EGF/PDGF*, and *WNT*) (Supplementary Table S2). We also explored whether EMT is associated with the invasiveness of PC-3 cells via IL-7 in the IL-7Rα^high^IL-7^high^ group. Stemness-related genes of both the CSC and EMT pathways were significantly enriched in the IL-7Rα^high^IL-7^high^ group compared with IL-7Rα^low^IL-7^low^ group (Fig. 5B), being consistent with proposed the mechanism involved in IL-7-induced tumor cell migration and invasion based on transcriptomic analysis of a human cancer samples. This result strongly suggests that IL-7/IL-7R enhances the invasiveness of prostate cancer in patients.

## Discussion

In this study, we modulated the expression of IL-7Rα and used inhibitors to show that IL-7 directly increases the migration and invasion of prostate cancer cells and assessed the potential of IL-7 and IL-7Rα as targets for anticancer drugs. We found that IL-7 induced the EMT in PC-3 cells, leading to increased cell invasion and bone metastasis. In particular, gene expression data for prostate cancer patient samples showed that expression of EMT- and stem cell-related genes was prominent in cancer cells highly expressing both IL-7 and IL-7Rα. These findings suggest that IL-7Rα plays a crucial role in the migration and invasion of prostate cancer cells and may represent a novel anticancer target.

In this study, we also found that DU-145 as well as PC-3 cells and its derivatives, known as androgen receptor (AR)-negative cells, express IL-7Rα, but not AR-positive prostate cancer cells such as VCaP and LNCaP-LN3 cells (Supplementary Fig. S6). We thought that IL-7Rα expression may be a characteristic of advanced prostate cancer (*i*.*e*., AR-independence). This raises the hypothesis that the androgen signal can suppress the expression of IL-7Rα in AR-positive prostate cancer cells because IL-7Rα is expressed only in AR-negative prostate cancer cells. However, bicalutamide, an AR blocker, did not increase the expression of IL-7Rα in AR-positive prostate cancer cells (Supplementary Fig. S7). Thus, we thought that we could not find the regulation of IL-7Rα expression by the AR blocker because we had limitation to mimic the *in vivo* environment sufficiently.

Although several studies have explored the role of IL-7 in tumors^6–8^, various cancer and prostate cancer cell lines have been evaluated, different from the cell lines used in this study. For example, Qu *et al*.^6^ showed that IL-7 promotes the migration and invasion of DU-145 prostate cancer cells via activation of the Akt/NF-κB pathway and upregulation of MMP3 and MMP7 expression. DU-145 cells, derived from brain metastasis, show moderate metastatic potential compared with the high metastatic potential of PC-3 cells^58^. Thus, this difference in metastatic potential suggests that the mechanism of response to IL-7 also differs. On the other hand, IL-7 promotes the proliferation of A549 and LH7 lung cancer cells by upregulating cyclin D1 via the c-Fos/c-Jun pathway^8^ and enhances the growth of MCF-7 and MDA MB-231 breast cancer cells via a Wortmannin-sensitive pathway^59^. However, the growth of PC-3 cells, *in vitro* and *in vivo*, was not affected by IL-7 (Supplementary Fig. S3A,B). In addition, although PC-3M and PC-3M-MM2 cells express IL-7Rα, the reactivity to IL-7 was different between these two cells and PC-3 cells, and the invasion ability was different according to *in vitro* ECM-mimicking environment (Fig. 1). PC-3M and PC-3M-MM2 were established from cells that metastasized in the liver^21^ and bone^22^, respectively, by transplanting PC-3 cells into nude mice, indicating that PC-3 cells isolated from humans became adapted to the mice. Thus, unlike PC-3, factors other than IL-7 may induce the invasion of PC-3M and PC-3M-MM2 cells in mice. This suggests that the reactivity to IL-7 may vary depending on the cancer origin or its derivatives.

Although EMT plays a role in embryonic development, inducing epithelial cell transformation into mesenchymal cells, EMT is also associated with tumor metastasis^44,60,61^. In this study, we found that IL-7 increased the EMT marker expressions of PC-3 cells, and this effect was specifically inhibited by the IL-7 blocking antibody M25 (Fig. 4B,C). Thus, although we believe that migration and invasion of PC-3 cells by IL-7 may be mediated by altered expression of EMT markers, further studies are required to identify the upstream signaling pathways that increase the IL-7-induced expression of EMT markers, and to determine whether IL-7Rα overexpression or IL-7R signaling inhibitors in PC-3 cells affect tumor metastasis, particularly the typical bone metastasis of prostate cancer *in vivo*. Furthermore, it is well known that the tumor microenvironment, especially the immune system, is a very important factor for tumor survival and metastasis^62^. Thus, the effect of IL-7 on the invasiveness of prostate cancer should be clarified through the orthotopic tumor model considering tumor microenvironment similar to that of the original tumor. However, since we did not establish a proper orthotopic tumor model yet, we would like to evaluate the effect of IL-7 on prostate cancer in the future.

In conclusion, the prognosis of prostate cancer is poor because there is no appropriate treatment for the subsequent bone metastasis of prostate cancer. Recently, in patients with a poor prognosis, it was shown that IL-7 is highly expressed, which affects cell proliferation and penetration in various solid tumors such as lung, breast, and bladder cancers^7,63^. These findings indicate that IL-7 and IL-7R play important roles in tumors and are promising markers indicating the presence of signaling molecules such as STAT5, which can be used to diagnose cancer patients at risk of a poor prognosis^15,16,64,65^. This study showed that IL-7 enhances the migration and invasion of prostate cancer cells via EMT, suggesting that IL-7 and IL-7Rα are therapeutic targets for the treatment of prostate cancer.

## Methods

### Cell culture

Prostate cancer cell lines PC-3^66^, PC-3M^21^, and PC-3M-MM2^22^ were obtained from the Korean Cell Line Bank (Seoul, Republic of Korea) and grown in RPMI 1640 medium containing 10% fetal bovine serum and antibiotics (*i*.*e*., RPMI complete medium, Life Technologies). IL-7Rα-knockdown PC-3 cells (PC-shIL7R) and IL-7Rα-overexpressing PC-3 cells (PC-IL7ROE) were established using lentiviral vectors, as described below. All the cell lines were authenticated and free of mycoplasma.

### Plasmid construction and lentiviral transduction

The lentiviral vector overexpressing IL-7Rα was constructed as previously described^67^. To construct the *IL7RA* shRNA-expressing lentiviral vector, the following oligonucleotides forming shRNAs were incorporated into the pLVX-shRNA2 lentiviral vector (Clontech Laboratories): 5′-GAT-CCG-GAA-GAG-ATT-CAT-CCC-TCA-TTC-AAG-AGA-TGA-GGG-ATG-AAT-CTC-TTC-CTT-TTT-TAC-GCG-TG-3′ and 5′-AAT-TCA-CGC-GTA-AAA-AAG-GAA-GAG-ATT-CAT-CCC-TCA-TCT-CTT-GAA-TGA-GGG-ATG-AAT-CTC-TTC-CG-3′. Lentiviruses were produced as previously described^67^. The results were confirmed by flow cytometry at 72 h post-transduction.

### Flow cytometry, cell sorting, and reagents

To measure the expression of IL-7Rα, cells were stained with Phycoerythrin-conjugated anti-human IL-7Rα Abs (BD Biosciences). The BD™ LSRII (BD Biosciences) ran the stained cells and the FlowJo® software (TreeStar) was used to analyze the data. The GFP-expressing PC-3 cells derivatives, PC-IL7ROE and PC-shIL7R, were sorted using the BD FACSAria™ (BD Biosciences).

The anti-human/mouse IL-7 monoclonal Ab M25 was kindly provided by Amgen Inc., and the JAK inhibitor tofacitinib was purchased from Sigma-Aldrich.

### Immunofluorescence staining

Cells were grown on 15-mm round coverslips (Neuvitro) for 18 h and treated with 2 mM monensin (GolgiStop®, BD Biosciences) for the last 4 h of the incubation. Cells were then fixed with 4% paraformaldehyde (PFA) for 15 min at room temperature. Subsequently, the cells were blocked with 1% rat normal serum in PBS for 1 h and incubated with anti-human IL-7 (Proteintech) or anti-IL-7Rα (Lifespan Biosciences) Abs at 4 °C for overnight. Next, the wells were incubated with Alexa Fluor® 488-anti-mouse Ig Abs and Alexa Fluor® 594-anti-rabbit Ig Abs, respectively. Stained cells were visualized under an Olympus confocal microscope (Olympus).

### Wound healing assay

Cells (5 × 10^5^) were cultured on 24-well plates until 95% confluency in 1 mL RPMI complete media. A wound was produced in the cell monolayer using tips. After washing the plates once with PBS, IL-7 (10 ng/mL, PeproTech) treatment was given at the appropriate times. Images of migrated cells were taken under microscope (×100). The distance of each scratch closure (indicating wound healing) was measured.

### *In vitro* invasion assay

Matrigel matrix solution (250 μg/mL, BD Biosciences) was applied on 8-μm microporous polycarbonate membranes in transwell chambers (BD Biosciences) and incubated at 37 °C for at least 5 h to allow gelling. Cells (1 × 10^5^) with or without IL-7 (10 ng/mL) were seeded on the upper chamber of the transwell, after collagen matrix (5 μg/mL) was applied on the lower chamber. Invasion assays were performed afterward, as previously described^46,47^. Images were taken using an inverted microscope (×200). Cell counts were done by ImageJ software (http://imagej.nih.gov/ij/, National Institute of Health).

The vertical collagen gel invasion assay was performed as described previously^68^. Briefly, a vertical collagen gel chamber was constructed using two gelatin-coated cover slides placed 1 mm apart facing one another, three sides of which were sealed with paraffin. A total of 400 μL liquid-phase collagen gel (type I collagen, 2 mg/mL, Nitta Gelain) was poured into the space between the two cover slides. After the gel was allowed to harden, Dil (Molecular Probes)-labeled cells (4 × 10^5^/100 μL), which were stimulated for 18 h with PBS or IL-7 (10 ng/mL), were seeded onto the top surface of the collagen gel and incubated at 37 °C for 18 h in 5% CO_2_ in a vertical position. The images of migrated cells were taken by fluorescence microscopy (×100). The migration of the cells was measured as the maximum distance or as the frequency of cells that migrated a certain distance from the surface of the collagen gel using ImageJ software.

### Sphere formation assay

To examine sphere forming capacity in response to IL-7, the sphere formation assay was performed as described previously^69^. Cells were seeded on nonadherent 96-well plates (Corning) at 2 × 10^2^/100 μL/well (n = 20) with or without IL-7 and maintained for one week. The medium was not changed or added so as to disturb the formation of sphere. Visible spheres (>100 μm in diameter) were counted under a microscope (×10) after 7-day incubation. For serial passages of the sphere, spheres were dissociated into single cells using enzymatic digestion with trypsin-EDTA (Life Technologies). The total number of spheres formed from 20 wells was counted each experiment, and the average for three independent experiments was calculated.

### Mouse model of prostate cancer

All experimental protocols were approved by the Institutional Animal Care and Use Committee (IACUC) of Seoul National University (Authorization No. SNU140716-1-1). All experiments were performed in accordance with the guidelines of the IACUC of Seoul National University. All mice used for this study were bred and housed under specific pathogen-free conditions in the animal facility of Seoul National University College of Medicine. Eight-week-old male athymic nude mice were purchased from the Envigo and maintained at the animal facility of Seoul National University. For a skeletal metastasis model, the procedure described by Park *et al*. was followed^55^. Briefly, 2 × 10^5^ cells were injected into the left heart ventricle of male athymic nude mice. Mice were sacrificed after 3 days, and whole hindlimb DNA was harvested in order to perform *Alu* and murine *β-actin* quantitative PCR^57^.

### Quantitative polymerase chain reaction (qPCR)

Total RNA extraction from cells stimulated for 24 h with IL-7 (10 ng/mL) in the presence of M25 or isotype control Abs (100 μg/mL) was done by TRIzol® (Life Technologies). cDNA was synthesized from the extracted RNA using the Transcript First Strand cDNA Synthesis Kit (Roche Applied Science). SYBR® Green Real-Time PCR Master Mix (Applied Biosystems) was used to perform PCR on the QuantStudio™ 6 Flex (Applied Biosystems) (*see* Table S1 for primer sequences). Glyceraldehyde 3-phosphate dehydrogenase (*GAPDH*) was used as a control gene to normalize differences in expression.

To quantify disseminated tumor cells, the genomic DNA was extracted immediately from the bone marrow from both hindlimbs using the DNeasy Blood and Tissue Kit (Qiagen), as previously described^56,57^. To determine the tumor cell-derived DNA in the bone marrow, 100 ng of genomic DNA was subjected to qPCR to detect the human *Alu* sequence. Each sample was normalized by murine *β-actin*. The primers used in qPCR were as follows: human *Alu* forward, 5′-GTC-AGG-AGA-TCG-AGA-CCA-TCC-T-3′, and reverse, 5′-AGT-GGC-GCA-ATC-TCG-GC-3′. The *Alu* signal was normalized against the relative quantity of murine *β-actin* and expressed as ΔCt (Ct_alu_ − Ct_β-actin_). The relative changes in metastasis were calculated as 2^−ΔCt^.

### Immunoblot analysis

Cells were starved for 24 h, followed by IL-7 (100 ng/mL) stimulation in the presence of M25 or isotype control Abs for the indicated times, and then subjected to immunoblotting to analyze IL-7R signaling molecules. To analyze EMT markers, the cells were stimulated for 72 h with IL-7 (10 ng/mL) in the presence of M25 or isotype control Abs (100 μg/mL). The immunoblotting used the following Abs: rabbit monoclonal IL-7 (Proteintech) and Snail Abs (Abcam); rabbit polyclonal STAT5, phospho (p)-STAT5 (Tyr694), p-Akt (Ser473), Akt, p-p44/42 mitogen-activated protein kinase (MAPK)/Erk (Thr202/Tyr204), Zeb1, and Erk Abs (all from Cell Signaling Technology); and mouse monoclonal N-cadherin, E-cadherin (both from Santa Cruz Biotechnology), vimentin (DAKO), and β-actin Abs (Sigma-Aldrich).

### Functional enrichment analysis

Transcriptomic data from 499 prostate cancer patients were obtained from TCGA-PRAD RNA-Seq data from NCI Genomic Data Commons, and the normalized read values (fragments per kilobase of exon per million reads) were log2 transformed. A manually curated set of 14,828 genes was obtained from the gene expression profile and used for further analysis.

Gene set enrichment analysis (GSEA) was used to identify genes enriched in either IL-7Rα^high^IL-7^high^ (defining the top 10% of the population) or IL-7Rα^low^IL-7^low^ (defining the lower 10% of the population) patients. The GSEA analysis tool from the Broad Institute website (http://www.broadinstitute.org/gsea/index.jsp) was used. Gene lists for the functional enrichment analysis were based on the Human RT^2^ Profiler PCR array (SABiosciences).

### Statistical analysis

All data were expressed as means ± standard error of the mean (SEM). Data comparison was done by the Mann–Whitney *U* test and one-way analysis of variance (ANOVA), followed by Bonferroni *post hoc* tests. The Pearson’s correlation analysis was performed between two variables. *P*-values < 0.05 were shown significant and all statistical analyses were done by GraphPad Prism 6.01 (GraphPad Software).

## Supplementary information

Supporting Information

## References

[1] Siegel RL, Miller KD, Jemal A (2017). Cancer Statistics, 2017. CA Cancer J Clin.

[2] Wingo PA, Tong T, Bolden S (1995). Cancer statistics, 1995. CA Cancer J Clin.

[3] Tharmalingam S, Chow E, Harris K, Hird A, Sinclair E (2008). Quality of life measurement in bone metastases: A literature review. J Pain Res.

[4] Saad F (2004). Long-term efficacy of zoledronic acid for the prevention of skeletal complications in patients with metastatic hormone-refractory prostate cancer. Journal of the National Cancer Institute.

[5] Schluns KS, Kieper WC, Jameson SC, Lefrancois L (2000). Interleukin-7 mediates the homeostasis of naive and memory CD8 T cells *in vivo*. Nat Immunol.

[6] Qu H (2016). IL-7/IL-7 receptor axis stimulates prostate cancer cell invasion and migration via AKT/NF-kappaB pathway. Int Immunopharmacol.

[7] Park SL, Lee EJ, Kim WJ, Moon SK (2014). p27KIP1 is involved in ERK1/2-mediated MMP-9 expression via the activation of NF-kappaB binding in the IL-7-induced migration and invasion of 5637 cells. Int J Oncol.

[8] Ming J, Jiang G, Zhang Q, Qiu X, Wang E (2012). Interleukin-7 up-regulates cyclin D1 via activator protein-1 to promote proliferation of cell in lung cancer. Cancer Immunol Immunother.

[9] Jiang Q (2005). Cell biology of IL-7, a key lymphotrophin. Cytokine Growth Factor Rev.

[10] Maeurer MJ (1997). Interleukin-7 (IL-7) in colorectal cancer: IL-7 is produced by tissues from colorectal cancer and promotes preferential expansion of tumour infiltrating lymphocytes. Scand J Immunol.

[11] Trinder P, Seitzer U, Gerdes J, Seliger B, Maeurer M (1999). Constitutive and IFN-gamma regulated expression of IL-7 and IL-15 in human renal cell cancer. Int J Oncol.

[12] Xie X (2004). Interleukin-7 and suppression of local peritoneal immunity in ovarian carcinoma. Int J Gynaecol Obstet.

[13] Lambeck AJ (2007). Serum cytokine profiling as a diagnostic and prognostic tool in ovarian cancer: a potential role for interleukin 7. Clin Cancer Res.

[14] Mengus C (2011). Elevated levels of circulating IL-7 and IL-15 in patients with early stage prostate cancer. J Transl Med.

[15] Li H (2004). Activation of signal transducer and activator of transcription 5 in human prostate cancer is associated with high histological grade. Cancer Res.

[16] Gu L (2010). Stat5 promotes metastatic behavior of human prostate cancer cells *in vitro* and *in vivo*. Endocr Relat Cancer.

[17] Roato I (2010). Bone invading NSCLC cells produce IL-7: mice model and human histologic data. BMC Cancer.

[18] Roato I (2006). IL-7 up-regulates TNF-alpha-dependent osteoclastogenesis in patients affected by solid tumor. PLoS One.

[19] Roato I (2008). Osteoclasts are active in bone forming metastases of prostate cancer patients. PLoS One.

[20] Kaighn ME, Narayan KS, Ohnuki Y, Lechner JF, Jones LW (1979). Establishment and characterization of a human prostatic carcinoma cell line (PC-3). Invest Urol.

[21] Kozlowski JM (1984). Metastatic behavior of human tumor cell lines grown in the nude mouse. Cancer Res.

[22] Delworth M (1995). Systemic administration of 4-amidinoindanon-1-(2′-amidino)-hydrazone, a new inhibitor of s-adenosylmethionine decarboxylase, produces cytostasis of human prostate-cancer in athymic nude-mice. Int J Oncol.

[23] Mazzucchelli R, Durum SK (2007). Interleukin-7 receptor expression: intelligent design. Nat Rev Immunol.

[24] McElroy CA (2012). Structural reorganization of the interleukin-7 signaling complex. Proc Natl Acad Sci USA.

[25] Crawley JB (1997). T cell proliferation in response to interleukins 2 and 7 requires p38MAP kinase activation. J Biol Chem.

[26] Fleming HE, Paige CJ (2001). Pre-B cell receptor signaling mediates selective response to IL-7 at the pro-B to pre-B cell transition via an ERK/MAP kinase-dependent pathway. Immunity.

[27] Kittipatarin C, Khaled AR (2007). Interlinking interleukin-7. Cytokine.

[28] Gao J, Zhao L, Wan YY, Zhu B (2015). Mechanism of Action of IL-7 and Its Potential Applications and Limitations in Cancer Immunotherapy. Int J Mol Sci.

[29] Kleinman HK, Martin GR (2005). Matrigel: Basement membrane matrix with biological activity. Seminars in Cancer Biology.

[30] Hanson DA (1992). A specific immunoassay for monitoring human bone resorption: quantitation of type I collagen cross-linked N-telopeptides in urine. J Bone Miner Res.

[31] Kern B, Shen J, Starbuck M, Karsenty G (2001). Cbfa1 contributes to the osteoblast-specific expression of type I collagen genes. J Biol Chem.

[32] Kremer JM (2009). The safety and efficacy of a JAK inhibitor in patients with active rheumatoid arthritis: Results of a double-blind, placebo-controlled phase IIa trial of three dosage levels of CP-690,550 versus placebo. Arthritis Rheum.

[33] Lee EB (2014). Tofacitinib versus methotrexate in rheumatoid arthritis. N Engl J Med.

[34] Meyer DM (2010). Anti-inflammatory activity and neutrophil reductions mediated by the JAK1/JAK3 inhibitor, CP-690,550, in rat adjuvant-induced arthritis. J Inflamm (Lond).

[35] Chambers AF, Matrisian LM (1997). Changing views of the role of matrix metalloproteinases in metastasis. J Natl Cancer Inst.

[36] Fingleton B (2003). Matrix metalloproteinase inhibitors for cancer therapy:the current situation and future prospects. Expert Opin Ther Targets.

[37] Nemeth JA (2002). Matrix metalloproteinase activity, bone matrix turnover, and tumor cell proliferation in prostate cancer bone metastasis. J Natl Cancer Inst.

[38] Parks WC, Wilson CL, Lopez-Boado YS (2004). Matrix metalloproteinases as modulators of inflammation and innate immunity. Nat Rev Immunol.

[39] Daja MM, Niu X, Zhao Z, Brown JM, Russell PJ (2003). Characterization of expression of matrix metalloproteinases and tissue inhibitors of metalloproteinases in prostate cancer cell lines. Prostate Cancer Prostatic Dis.

[40] Lynch CC (2005). MMP-7 promotes prostate cancer-induced osteolysis via the solubilization of RANKL. Cancer Cell.

[41] Pivetta, E. S., M. MMP-13 stimulates osteoclast differentiation and activation in tumour breast bone metastases. *Breast Cancer Research* (2011). 10.1186/bcr3047 PMC3262218 22032644

[42] Casimiro S (2013). RANKL/RANK/MMP-1 molecular triad contributes to the metastatic phenotype of breast and prostate cancer cells *in vitro*. PLoS One.

[43] Polyak K, Weinberg RA (2009). Transitions between epithelial and mesenchymal states: acquisition of malignant and stem cell traits. Nat Rev Cancer.

[44] Thiery JP (2002). Epithelial-mesenchymal transitions in tumour progression. Nat Rev Cancer.

[45] Mani SA (2008). The epithelial-mesenchymal transition generates cells with properties of stem cells. Cell.

[46] Oh, K., Lee, O. Y., Park, Y., Seo, M. W. & Lee, D. S. IL-1 beta induces IL-6 production and increases invasiveness and estrogen-independent growth in a TG2-dependent manner in human breast cancer cells. *Bmc Cancer* **16**, 10.1186/s12885-016-2746-7 (2016). 10.1186/s12885-016-2746-7 PMC5017052 27609180

[47] Zhong Y (2010). Spheres derived from the human SK-RC-42 renal cell carcinoma cell line are enriched in cancer stem cells. Cancer Lett.

[48] Serrano-Gomez, S. J., Maziveyi, M. & Alahari, S. K. Regulation of epithelial-mesenchymal transition through epigenetic and post-translational modifications. *Molecular Cancer* **15**, 10.1186/s12943-016-0502-x (2016). 10.1186/s12943-016-0502-x PMC4765192 26905733

[49] Kalluri R, Weinberg RA (2009). The basics of epithelial-mesenchymal transition. J Clin Invest.

[50] Krebs AM (2017). The EMT-activator Zeb1 is a key factor for cell plasticity and promotes metastasis in pancreatic cancer. Nat Cell Biol.

[51] Jia D (2017). Distinguishing mechanisms underlying EMT tristability. Cancer Converg.

[52] Weitzenfeld P, Meshel T, Ben-Baruch A (2016). Microenvironmental networks promote tumor heterogeneity and enrich for metastatic cancer stem-like cells in Luminal-A breast tumor cells. Oncotarget.

[53] Zheng X (2015). Epithelial-to-mesenchymal transition is dispensable for metastasis but induces chemoresistance in pancreatic cancer. Nature.

[54] Moreno-Bueno G, Portillo F, Cano A (2008). Transcriptional regulation of cell polarity in EMT and cancer. Oncogene.

[55] Park, S. I., Kim, S. J., McCauley, L. K. & Gallick, G. E. Pre-clinical mouse models of human prostate cancer and their utility in drug discovery. *Curr Protoc Pharmacol* Chapter 14, Unit 14 15, 10.1002/0471141755.ph1415s51 (2010). 10.1002/0471141755.ph1415s51 PMC3072580 21483646

[56] Campbell, J. P. *et al*. TRIzol and Alu qPCR-based quantification of metastatic seeding within the skeleton. *Scientific Reports* **5**, 10.1038/srep12635 (2015). 10.1038/srep12635 PMC4536516 26271202

[57] Funakoshi K (2017). Highly sensitive and specific Alu-based quantification of human cells among rodent cells. Sci Rep.

[58] Pulukuri SM (2005). RNA interference-directed knockdown of urokinase plasminogen activator and urokinase plasminogen activator receptor inhibits prostate cancer cell invasion, survival, and tumorigenicity *in vivo*. J Biol Chem.

[59] Al-Rawi MA, Rmali K, Mansel RE, Jiang WG (2004). Interleukin 7 induces the growth of breast cancer cells through a wortmannin-sensitive pathway. Br J Surg.

[60] Kang Y, Massague J (2004). Epithelial-mesenchymal transitions: twist in development and metastasis. Cell.

[61] Ota I (2016). Snail-induced EMT promotes cancer stem cell-like properties in head and neck cancer cells. Oncol Rep.

[62] Quail DF, Joyce JA (2013). Microenvironmental regulation of tumor progression and metastasis. Nat Med.

[63] Al-Rawi MA, Rmali K, Watkins G, Mansel RE, Jiang WG (2004). Aberrant expression of interleukin-7 (IL-7) and its signalling complex in human breast cancer. Eur J Cancer.

[64] Al-Rawi MAA, Mansel RE, Jiang WG (2003). Aberrant expression of interleukin-7 (IL-7) and its receptor (IL-7R) in breast cancer and the association with prognosis. British Journal of Cancer.

[65] Schroten C (2012). The additional value of TGFbeta1 and IL-7 to predict the course of prostate cancer progression. Cancer Immunol Immunother.

[66] Janssen T (1996). *In vitro* characterization of prolactin-induced effects on proliferation in the neoplastic LNCaP, DU145, and PC3 models of the human prostate. Cancer.

[67] Kim JH (2017). Interleukin-7 Induces Osteoclast Formation via STAT5, Independent of Receptor Activator of NF-kappaB Ligand. Front Immunol.

[68] Cho Y (2010). Adipokine Resistin Is a Key Player to Modulate Monocytes, Endothelial Cells, and Smooth Muscle Cells, Leading to Progression of Atherosclerosis in Rabbit Carotid Artery. Journal of the American College of Cardiology.

[69] Oh, K. *et al*. Transglutaminase 2 facilitates the distant hematogenous metastasis of breast cancer by modulating interleukin-6 in cancer cells. *Breast Cancer Research* **13**, 10.1186/bcr3034 (2011). 10.1186/bcr3034 PMC3262209 21967801

