# Supplementary material for: Interleukin-7 Contributes to the Invasiveness of Prostate Cancer Cells by Promoting Epithelial–Mesenchymal Transition
Source: Sci Rep. 2019 May 6;9:6917. doi: 10.1038/s41598-019-43294-4 (PMC6502845; doi:10.1038/s41598-019-43294-4)
Supplement: Supplementary file 1 — Supporting Information [file 41598_2019_43294_MOESM1_ESM.pdf]

Interleukin-7 Contributes to the Invasiveness of Prostate Cancer Cells by Promoting Epithelial–Mesenchymal Transition

Min A Seol[1,2†], Jin-Hee Kim[1,7†], Keunhee Oh[1,2†], Gwanghun Kim[1,2,3], Myung Won Seo[1], Young-Kyoung Shin[4], Ji Hyun Sim[1], Hyun Mu Shin[1,2,3,6], Bo Yeon Seo[9,10], Dong-Sup Lee[1,2,3], Ja-Lok Ku[2,3,4], Ilkyu Han[5], Insoo Kang[8], Serk In Park[9,10,11], and Hang-Rae Kim[1,2,3,6,8*]

[1]Department of Anatomy and Cell Biology, [2]Department of Biomedical Sciences, [3]BK21Plus Biomedical Science Project, [4]Cancer Research Institute, [5]Department of Orthopedic Surgery, and [6]Medical Research Institute, Seoul National University College of Medicine, Seoul 03080, Republic of Korea

[7]Department of Biomedical Laboratory Science, College of Health Science, Cheongju University, Cheongju, Chungbuk 28150, Republic of Korea

[8]Department of Internal Medicine, Section of Rheumatology, Yale University School of Medicine, New Haven, CT 06520 USA.

[9]Department of Biochemistry and Molecular Biology and [10]BK21 Plus Program, Korea University College of Medicine, Seoul 02841, Republic of Korea.

[11]Department of Medicine, Vanderbilt University School of Medicine, Nashville, TN 37232 USA.

†These authors contributed equally

Supplementary Figures

Figure S1

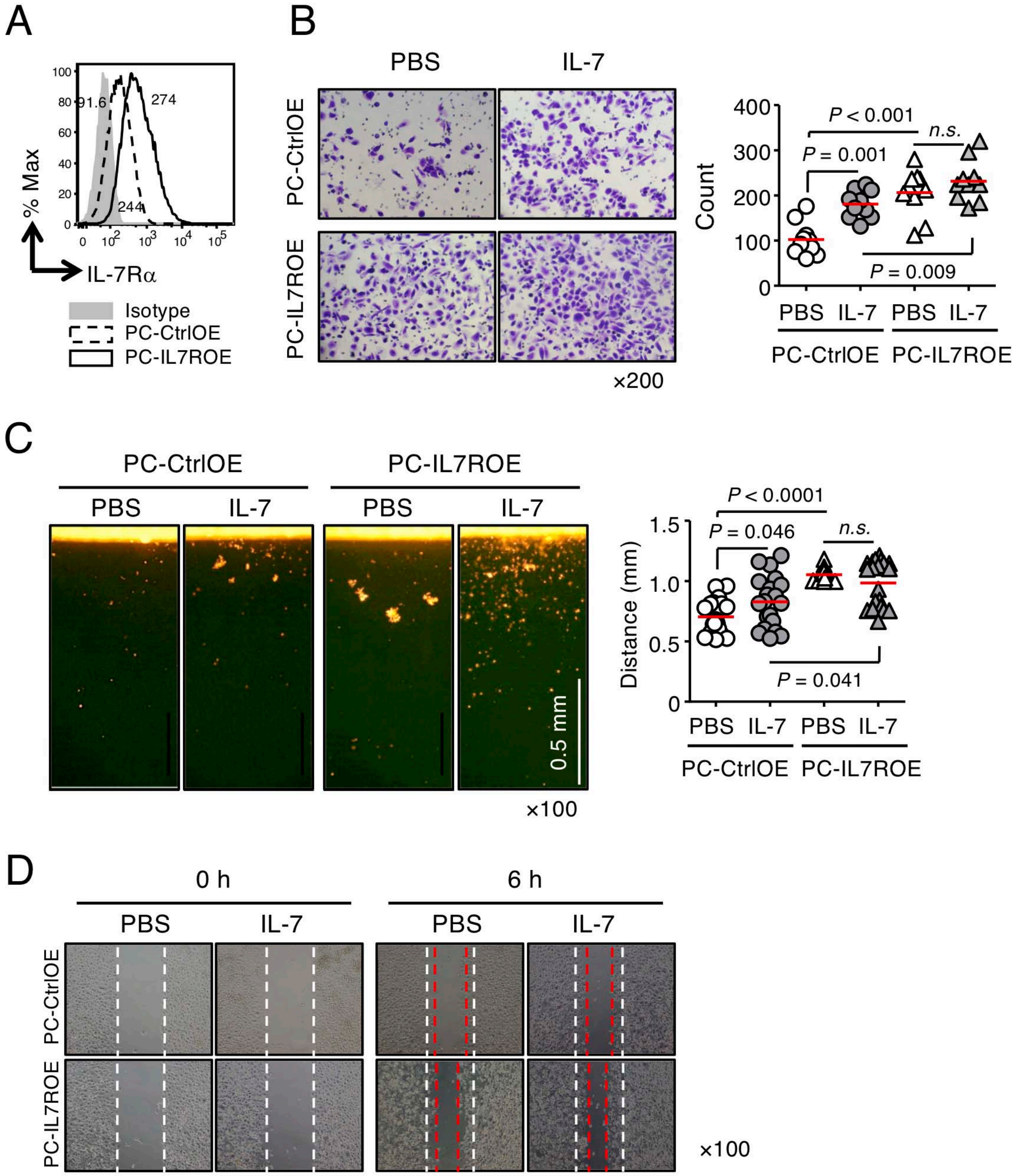

Figure S1. **Cells overexpressing IL-7Rα exhibited enhanced migration and invasion compared with the control cells.** (**A**) IL-7Rα-overexpressing PC-3 (PC-IL7ROE) and control

cells (PC-CtrlOE) were established by lentiviral transduction. Cells were stained with anti-IL-7Rα Abs and analyzed by flow cytometry. The numbers in the histogram indicate the mean fluorescence intensity. (**B**) Cells were allowed to invade through matrigel (250 μg/mL) for 24 h in the presence or absence of IL-7 (10 ng/mL). Invading cells were stained with crystal violet (left panel), counted using ImageJ software, and plotted as a graph (right panel). (**C**) Dil-labeled cells were allowed to invade through vertical collagen gel (2 mg/mL) for 18 h in the presence or absence of IL-7 (10 ng/mL). Representative image of cells invading vertical collagen gel (left panel) and dot graph showing the maximum distances of cell invasion (right panel). Scale bar = 0.5 mm. (**D**) Wound-healing migration of PC-IL7ROE and control cells was performed after IL-7 (10 ng/mL) treatment for 6 h. Dashed lines indicate the initial boundaries of the scratches (white dotted lines, 0 h) and the cell leading edges at subsequent times (red dotted lines, 6 h). Bars indicate the means. *P*-values were obtained using the Mann–Whitney *U* test (**B–C**). *n.s.*, not significant. Results are representative of two or three independent experiments.

Figure S2

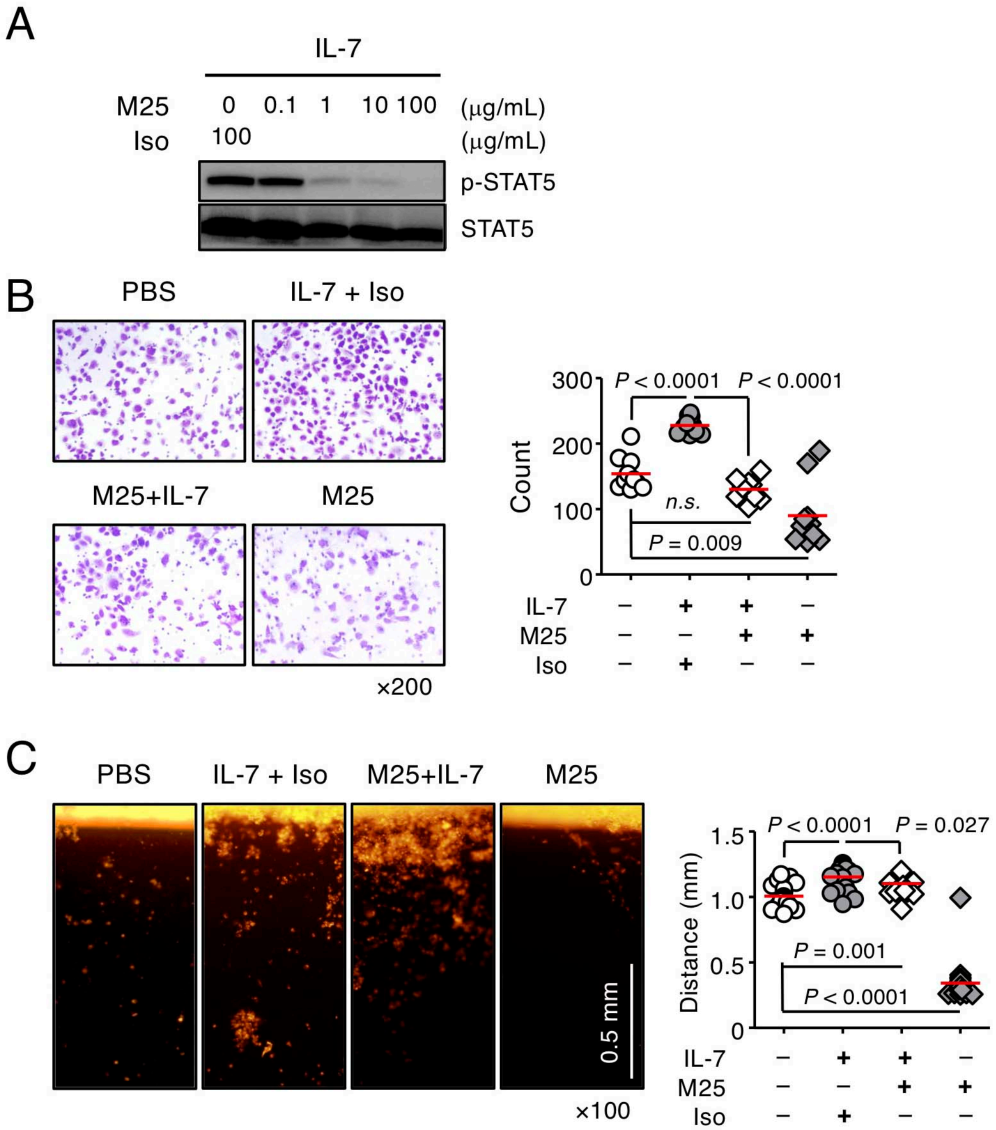

Figure S2. **The anti-IL-7 Ab M25 inhibited the invasiveness of PC-3 cells by IL-7.** (**A**) IL-7 (100 ng/mL)-stimulated PC-3 cells were treated with M25 at the indicated concentrations for 30 min and then subjected to immunoblot analysis of STAT5 phosphorylation. (**B**) PC-3 cells were allowed to invade through matrigel (250 μg/mL) for 24 h after treatment with IL-7 (10 ng/mL) in the presence of M25 or isotype control Abs (Iso) (100 μg/mL). Invading cells were stained with

crystal violet (left panel), counted using ImageJ software, and plotted as a graph (right panel). (**C**) DiI-labeled cells invaded vertical collagen gel (2 mg/mL) for 18 h after treatment with IL-7 (10 ng/mL) in the presence of M25 or isotype control Abs (Iso) (100 μg/mL). A representative image of cells invading vertical collagen gel (left panel) and dot graph showing the maximum distances of the invading cells (right panel). Scale bar = 0.5 mm. Bars indicate means. *P*-values were obtained using the Mann–Whitney *U* test (**B**–**C**). Results are representative of two or three independent experiments.

Figure S3

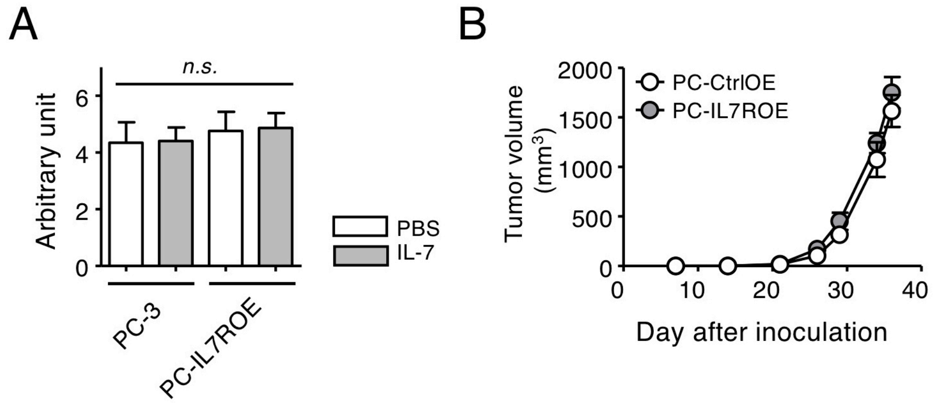

Figure S3. **Effect of IL-7 on the growth of prostate cancer cells *in vitro* and *in vivo.*** (**A**) Cells were cultured in the presence or absence of IL-7 (10 ng/mL) for 24 h and subjected to MTT assays. Results are the averages from two or three independent experiments. (**B**) Six- to eight-week-old male NSG mice were injected subcutaneously with 5×10^5 cells (100 μL). Tumor volume was determined from 1 week after implantation of tumor cells, PC-IL7ROE (n = 4), and control cells (n = 5). Bar and line graphs show the means ± standard error of the mean. *P*-values were obtained by one-way ANOVA of multiple comparisons. *n.s.*, not significant.

Figure S4

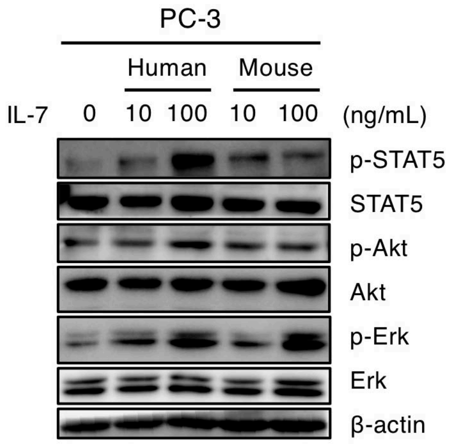

Figure S4. **Cross-reactivity of human and mouse IL-7 to PC-3 cells.** PC-3 cells were stimulated with recombinant human and mouse IL-7 and subjected to immunoblot analysis of phosphorylated STAT5, Akt, and Erk. Results are representative of two independent experiments.

Figure S5

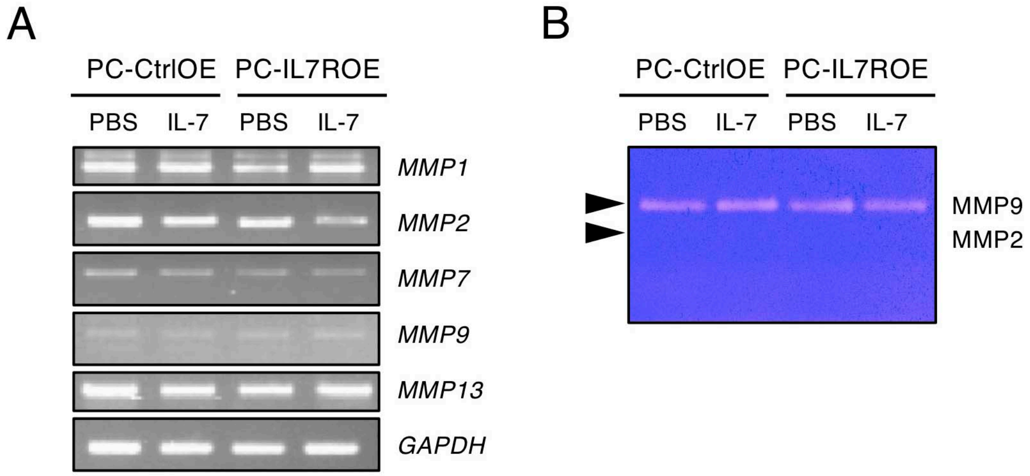

Figure S5. **Transcript levels and enzymatic activities of MMPs in PC-IL7ROE and control cells treated with IL-7.** (**A**) Transcript levels of *MMP1, MMP2, MMP7, MMP9,* and *MMP13* were determined by RT-PCR. (**B**) The levels of gelatinase were measured by gelatin zymography. Results are representative of two independent experiments.

Figure S6

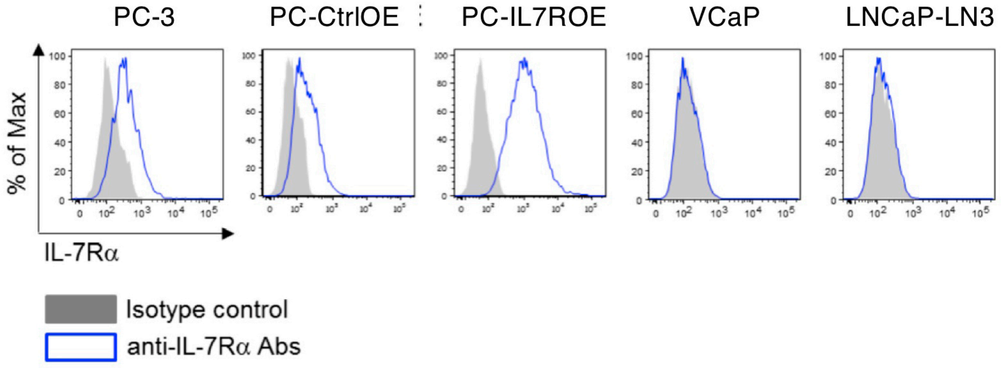

Figure S6. **IL-7Rα expression in androgen receptor (AR)-positive prostate cancer cells.** PC-3, IL-7Rα-overexpressing PC-3 (PC-IL7ROE) and control (PC-CtrlOE), AR-positive VCaP and LNCaP-LN3 cells were stained with anti-IL-7Rα Abs and analyzed by flow cytometry. Results are representative of two independent experiments.

Figure S7

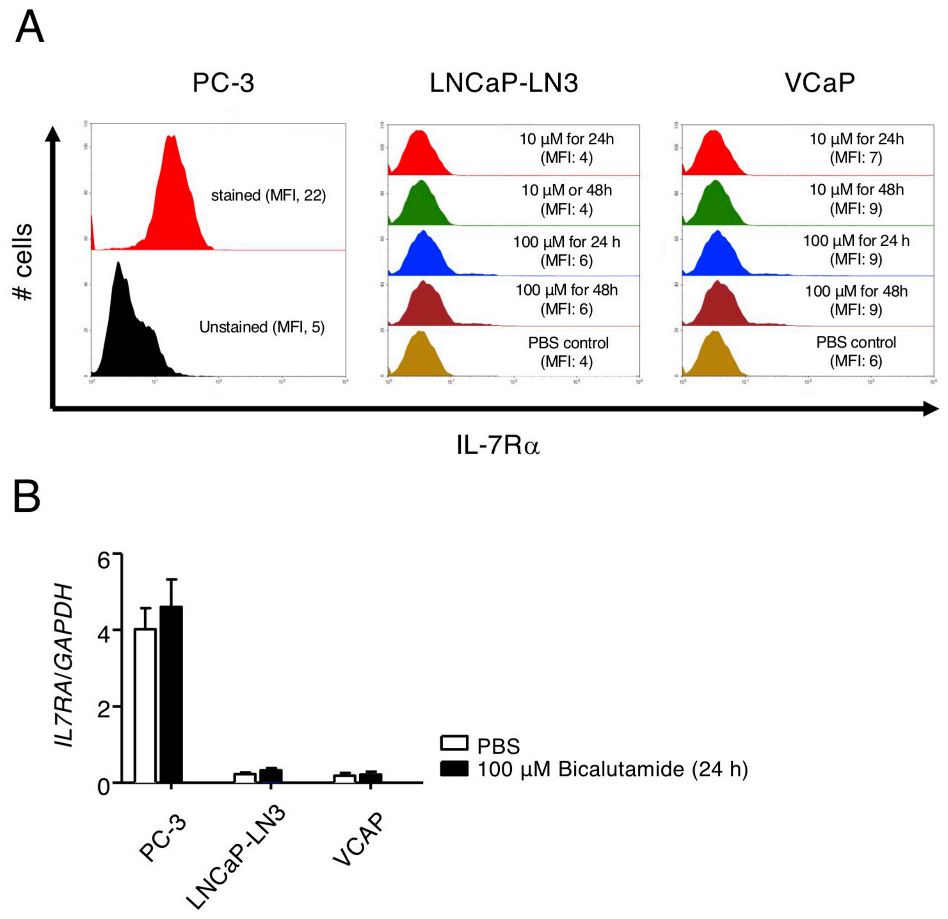

Figure S7. **AR blocker did not change the expression of IL-7Rα in AR-positive prostate cancer cell lines.** (**A-B**) Translational (**A**) and transcriptional (**B**) levels of IL-7Rα in AR-positive prostate cancer cells in response to bicalutamide, an AR blocker, were analyzed. Bar graph shows the means ± standard error of the mean. Results are representative of two independent experiments.

Supplementary Tables

Table S1. Sequences of the PCR primers

Genes		Sequences
ZEB1	Forward	5’-GGG-AGG-AGC-AGT-GAA-AGA-GA-3’
	Reverse	5’-TTT-CTT-GCC-CTT-CCT-TTC-TG-3’
ZEB2	Forward	5’-AAG-CCA-GGG-ACA-GAT-CAG-C-3’
	Reverse	5’-CCA-CAC-TCT-GTG-CAT-TTG-AAC-T-3’
TWIST1	Forward	5’-AAG-GCA-TCA-CTA-TGG-ACT-TTC-TCT-3’
	Reverse	5’-GCC-AGT-TTG-ATC-CCA-GTA-TTT-T-3’
SNAI1	Forward	5’-CCA-CAC-TGG-TGA-GAA-GCC-A -3’
	Reverse	5’-TCT-TCA-CAT-CCG-AGT-GGG-TTT-G -3’
SNAI2	Forward	5’-TGG-TTG-CTT-CAA-GGA-CAC-AT-3’
	Reverse	5’-GTT-GCA-GTG-AGG-GCA-AGA-A-3’
MMP1	Forward	5’-GAT-GGG-AGG-CAA-GTT-GAA-AA-3’
	Reverse	5’-CTG-CTT-GAC-CCT-CAG-AGA-CC-3’
MMP2	Forward	5’-CAC-TTT-CC-TGG-GCA-ACA-AAT-3’
	Reverse	5’-GCC-TCG-TAT-ACC-GCA-TCA-AT-3’
MMP7	Forward	5’-TGT-ATG-GGG-AAC-TGC-TGA-CA-3’
	Reverse	5’-TGG-GGA-TCT-CCA-TTT-CCA-TA-3’
MMP9	Forward	5’-GGC-GCT-CAT-GTA-CCC-TAT-GT-3’
	Reverse	5’-TCA-AAG-ACC-GAG-TCC-AGC-TT-3’
MMP13	Forward	5’-GTG-GTG-TGG-GAA-GTA-TCA-TCA-3’
	Reverse	5’- GCA-TCT-GGA-GTA-ACC-GTA-TTG-3’
IL7RA	Forward	5’-TGG-ACG-CAT-GTG-AAT-TTA-TC-3’
	Reverse	5’-ATT-CAC-TCC-AGA-AGC-CTT-T-3’
GAPDH	Forward	5’-GCC-ACC-CAG-AAG-ACT-GTG-GA-3’
	Reverse	5’-TGT-CTG-GCG-TTT-TTG-GAT-GT-3’
ACTB	Forward	5’- GGC TGT-ATT-CCC-CTC-CAT-CG-3’
	Reverse	5’-CCA-GTT-GGT-AAC-AAT-GCC ATG-T-3’

Table S2. Gene sets significantly enriched, with the highest normalized enrichment scores, in IL-7Rα^{high}IL-7^{high} compared with IL-7Rα^{low}IL-7^{low} prostate cancer patients

	Terms	Sizes	ES	NES	*P*-value	FDR
1	Cell motility	81	0.57	1.62	2.00E-02	3.70E-02
2	Liver cancer	80	0.58	1.63	5.00E-03	3.80E-02
3	TNF signaling pathway	67	0.55	1.64	1.50E-02	3.80E-02
4	Inflammatory response & autoimmunity	50	0.80	1.63	0.00E+00	3.90E-02
5	Cancer stem cells	72	0.64	1.63	2.00E-03	3.90E-02
6	Osteogenesis	67	0.70	1.64	2.00E-03	3.90E-02
7	Insulin resistance	66	0.58	1.62	6.00E-03	3.90E-02
8	Osteoporosis	58	0.66	1.67	0.00E+00	4.00E-02
9	Aging	79	0.55	1.64	1.10E-02	4.00E-02
10	Type I interferon response	68	0.70	1.61	1.80E-02	4.10E-02
11	Leukemia	72	0.62	1.60	8.00E-03	4.20E-02
12	NF-κB signaling targets	67	0.73	1.60	8.00E-03	4.20E-02
13	Epithelial to mesenchymal transition	77	0.64	1.59	5.00E-03	4.40E-02
14	Focal adhesions	79	0.59	1.59	2.20E-02	4.50E-02
15	Endothelial cell biology	73	0.69	1.57	6.00E-03	4.60E-02
16	Stem cell signaling	79	0.58	1.57	1.50E-02	4.80E-02
17	Lung cancer	59	0.59	1.56	1.00E-02	5.00E-02
18	IL6/STAT3 signaling pathway	61	0.68	1.70	0.00E+00	5.10E-02
19	Tumor metastasis	72	0.56	1.54	1.50E-02	5.40E-02
20	NF-κB signaling pathway	71	0.63	1.71	3.00E-03	5.50E-02
21	Mesenchymal stem cells	64	0.62	1.55	1.50E-02	5.50E-02
22	EGF/PDGF signaling pathway	81	0.46	1.54	2.80E-02	5.50E-02
23	Angiogenesis	68	0.68	1.54	1.20E-02	5.60E-02
24	Angiogenic growth factors	52	0.70	1.53	1.00E-02	5.70E-02
25	Protease activated receptor signaling	68	0.65	1.53	1.70E-02	5.70E-02
26	Apoptosis	68	0.50	1.71	7.00E-03	5.80E-02
27	Extracellular matrix & adhesion molecules	73	0.70	1.53	2.20E-02	5.80E-02
28	Inflammasomes	62	0.62	1.78	3.00E-03	5.90E-02
29	Prostate cancer	77	0.51	1.52	1.40E-02	5.90E-02
30	Cystic fibrosis	68	0.62	1.52	3.00E-02	5.90E-02
31	Necrosis	70	0.49	1.73	3.00E-03	6.10E-02
32	WNT signaling pathway	71	0.51	1.51	2.00E-02	6.10E-02
33	JAK/STAT signaling pathway	76	0.62	1.76	0.00E+00	6.20E-02
34	Wound healing	65	0.70	1.51	7.00E-03	6.20E-02
35	WNT signaling targets	68	0.65	1.50	1.20E-02	6.40E-02
36	Estrogen receptor signaling	77	0.59	1.50	2.10E-02	6.40E-02
37	Hematopoiesis	66	0.72	1.73	0.00E+00	6.60E-02
38	Multiple sclerosis	70	0.69	1.79	0.00E+00	7.10E-02

39	Oxidative stress	73	0.50	1.46	2.10E-02	8.00E-02
40	Stem cell transcription factors	44	0.57	1.46	3.80E-02	8.00E-02
41	Breast cancer	79	0.54	1.46	3.20E-02	8.10E-02
42	Retinoic acid signaling	48	0.58	1.43	2.50E-02	9.10E-02

ES = enrichment score, NES = normalized enrichment score, FDR = false discovery rate. Positive enrichment scores correspond to enrichment in the IL-7Rα^{high}IL-7^{high} group. Negative enrichment scores correspond to enrichment in the IL-7Rα^{low}IL-7^{low} group.

Supplementary Materials and Methods

In vitro cell growth assay

Cells were seeded into 96-well plates at a density of 1×10^3/well in 100 μL and treated with IL-7 (10 ng/mL) for 24 h. MTT assay was performed at 4 h before the end of the incubation; 20 μL MTT solution (5 mg/mL in PBS) were added to each well, and the cells were incubated at 37°C for 4 h. The medium was removed, and 150 μL DMSO were added to each well. The plate was gently rotated on an orbital shaker for 10 min to dissolve the precipitate completely. The absorbance was measured at 560 nm using a microplate reader (Molecular Devices, Sunnyvale, CA, USA).

In vivo tumor growth

NOD/SCID/IL-2Rγ-null (NSG) mice were purchased from the Jackson Laboratory and maintained at the animal facility of Seoul National University College of Medicine. Experiments involving mice were approved by the Institutional Animal Care and Use Committee of Seoul National University (authorization no. SNU140716-1-1).

To determine *in vivo* tumor growth, 6–8-week-old male NSG mice were injected subcutaneously with 5×10^5 cells in 100 μL PBS using a 29-gauge insulin needle. Tumor volume was measured *in vivo* using an external caliper from 1 week after implantation of the tumor cells.

RT-PCR

For semi-quantitative RT-PCR, total RNA was extracted using TRIzol® (Life Technologies) from cells stimulated with or without IL-7 (10 ng/mL) for the indicated times. cDNA was synthesized from the extracted RNA using the Transcript First Strand cDNA Synthesis Kit

(Roche Applied Science). PCR was performed using the AccuPower® PCR PreMix (Bioneer) (*see* Table S1 for primer sequences) and a conventional PCR System (Bioneer). The amplified PCR product was resolved on an agarose gel stained with ethidium bromide. All samples were first adjusted to contain equal input amounts of *GAPDH* cDNA for semi-quantitative PCR.

Gelatin zymography

To determine the amounts of MMP2 and MMP9 secreted into the culture medium, zymography was used for semi-quantitative analysis of gelatinase levels. Equal aliquots of conditioned culture media from an equal number of cells were electrophoresed on a polyacrylamide gel containing 1 mg/mL gelatin. After electrophoresis, the gel was incubated in renaturing buffer (50 mM Tris-HCl [pH 7.4], 2.5% (v/v) Triton X-100) for 60 min at 37°C and subsequently incubated in developing buffer (50 mM Tris-HCl [pH 8.0], 150 mM NaCl, 10 mM $CaCl_2$, 0.02% (w/v) sodium azide) for 18 h at 37°C. Proteolytic bands were visualized by staining with 0.05% (w/v) Coomassie Brilliant Blue solution.

***In vitro* Stimulation of androgen receptor blocker**

Androgen receptor (AR)-positive human prostate cancer cell lines VCaP and LNCaP-LN3 were obtained from the Korean Cell Line Bank and grown in RPMI complete medium. To examine the IL-7Rα expression in response to an AR blocker, cells were stimulated in the presence of bicalutamide (Sigma-Aldrich) or PBS control for the indicated times. The expression of IL-7Rα was analyzed by quantitative RT-PCR (Applied Biosystems) and flow cytometry (BD Biosciences) by staining cells with Phycoerythrin-conjugated anti-human IL-7Rα Abs (BD Biosciences). The relative gene expression levels of *IL7RA* were normalized to *GAPDH*.
